# Increasing safer sexual behavior among Lao kathoy through an integrated social marketing approach

**DOI:** 10.1186/1471-2458-11-872

**Published:** 2011-11-16

**Authors:** Kim Longfield, Xouchai Panyanouvong, Judy Chen, Megan B Kays

**Affiliations:** 1 Population Services International, 1120 Nineteenth Street NW, Suite 600, Washington, DC 20036, USA; 2 Previous address: Population Services International, Laos; 3 Political and Economic Section, Australian Embassy, Vientiane, Laos

## Abstract

**Background:**

Although HIV prevalence has remained low in Laos thus far, there is reason to be concerned that Lao male-to-female (MtF) transgender persons (*kathoy*) and their partners may facilitate the spread of HIV. Little is known about how to most effectively reach kathoy with HIV prevention programming. This paper evaluates an intervention with Lao kathoy with the objective of increasing safe sex with regular and casual partners.

**Methods:**

Quantitative surveys were administered in November 2004 (*n *= 288) and June 2006 (*n *= 415) using time location sampling at venues where kathoy were known to congregate. Respondents were aged 15-35 and from three urban centers in Laos. UNIANOVA tests were used to compare baseline and follow-up survey data and to evaluate the impact of PSI's kathoy-specific interventions on items that changed significantly over time.

**Results:**

Exposure to the intervention was associated with higher levels of condom use at last anal sex with casual partners and greater use of water-based lubricant. Exposure was also linked to improved perceptions of product availability for condoms and water-based lubricant. Knowledge about the importance of consistent condom use improved over time as well as the need to use condoms with regular partners. Some HIV knowledge decreased over time and the intention to use condoms with casual partners when water-based lubricant is available also declined.

**Conclusions:**

Study results demonstrate the feasibility of reaching kathoy with an integrated social marketing approach; combining product promotion, peer education, and other types of interpersonal communication. The approach was successful at increasing condom use with casual partners and water-based lubricant use, but the importance of using condoms along with water-based lubricant must be emphasized and modified strategies are required for improving condom use with boyfriends. Future messages should emphasize consistent condom use with all types of partners as well as improve knowledge and correct misconceptions about HIV and AIDS, STIs, condom use, and lubricant use. It is also important that authorities create an enabling environment to support such interventions and help foster behavior change.

## Background

Laos has one of Southeast Asia's lowest national HIV prevalence rates, with less than 0.2% of the general population aged 15 to 49, or an estimated 6,000 persons, living with HIV [1]. Expansion of the epidemic remains a concern, however, due to high rates of sexually transmitted infections (STIs) among key populations at risk as well as high levels of cross-border migration with Laos' higher HIV-prevalence neighbors, Cambodia and Thailand [2].

Given that studies in Cambodia, Thailand, Vietnam, and other Asian countries have found high HIV prevalence among men who have sex with men (MSM), there is reason to be concerned about the potential for HIV to spread through Lao MSM networks. In early 2009, the first published study of HIV prevalence among Lao MSM found an HIV prevalence rate of 5.6% among 540 men in the capital city of Vientiane [3].

Male-to-female (MtF) transgender persons, known locally as "*kathoy*," constitute an MSM subgroup that warrants special attention in Laos. In this paper, the term "kathoy" refers to biological males who self-identify as female, though in appearance they may be either male or female. On the basis of their behavior, this paper discusses kathoy as a subcategory of MSM. It is important to note, however, that kathoy themselves do not usually consider themselves to be MSM in keeping with their female self-identity.

Little HIV-related data has been collected on Lao kathoy, but evidence from other Asian countries suggests that they could potentially be among the nation's groups at highest risk. Studies of Thai kathoy and Indonesian MtF transgender persons (*warias*) found HIV rates ranging from 12% to 22% [4,5].

Transgender persons tend to be politically disenfranchised and subject to varying levels of stigma related to their unique position in society [6]. Such challenges are linked to an increased risk for HIV infection. The psychosocial consequences of stigma can include depression and poor self-esteem, which contribute to low negotiation power in sexual relationships and low self-efficacy to negotiate condom use [7-11]. Additionally, transgender persons may engage in casual sex with multiple partners to affirm their female gender identities and attractiveness to men [11].

Lao kathoy's HIV risk behaviors have not been investigated extensively. The aforementioned MSM cohort study, which included 66 kathoy, reported a 14.4% consistent condom use rate with regular partners in the last 3 months and a 24.1% consistent condom use rate with non-regular partners in the last 3 months [3]. Studies from other Asian countries reveal similarly low levels of condom use. Two studies among Indonesian warias found that the self-reported condom use was an average of 1.2 during their last 5 sexual acts [12,13]. Subsequent studies have shown varying rates, with 12% to 41% of warias reporting consistent condom use [5,14]. MtF transgender persons in India and Bangladesh also had low levels of consistent condom use. [15,16] Cambodian MSM populations that included MtF transgender persons appeared to have higher levels of condom use, with only 13% reporting unprotected penetrative sex in the last month [17].

Inconsistent condom use and other risky sexual behaviors among MSM have been associated with poor HIV knowledge and low perceived threat of HIV infection. A study of Thai MSM, including kathoy, found that believing myths about the source of HIV infection and feeling that HIV is not very serious were associated with inconsistent condom use [18]. Among Indonesian warias, 40% of those who felt that they were not at risk for HIV had eight or more sexual partners per week [12]. A qualitative study of Lao kathoy found that while most were aware of HIV, some held contradictory beliefs about its transmission. Also, knowledge about HIV did not appear to translate into protective behaviors [19].

Kathoys' partners are a potential "bridge" population for HIV transmission into the general population. A survey of Lao men found that 18.5% reported having had sex with other men, including kathoy, and that most of this group also reported having sex with women [20]. Other research suggests that a small minority of Lao kathoy report having female partners [19]. The partners of Lao kathoy are typically young men who self-identify as heterosexual and have sexual relations with women, but also have sex with kathoy for sexual experience, pleasure, experimentation, or money [22]. Laos appears to be unlike other settings where transgender persons report high rates of involvement in commercial and transactional sex due to a lack of other opportunities for employment [5,7,8,21]; conversely, Lao kathoy report paying their male partners for sex [22].

In 2006, the Laos government took the unusual step of including kathoy in its *National Strategy and Action Plan on HIV/AIDS/STI 2006-2010*. It identified kathoy as subgroup of MSM, and called for "evidence-based information on MSM and kathoy to be available and programmatically used" [23]. The Laos Action Plan is a commendable effort that helps create an enabling environment for HIV prevention programming among kathoy. It does, however, reveal the tendency of public health agencies to treat MSM as a homogenous group that shares the same identity, risk factors, behaviors, and prevention programming preferences. MtF transgender persons may not identify with HIV prevention programs targeting MSM and neither may their partners. Many partners do not self-identify as gay or bisexual, nor do they consider having sex with MtF transgender persons a homosexual act [6]. These are important considerations to keep in mind when developing interventions that target kathoy with HIV prevention messages.

To evaluate the effect of its kathoy-specific social marketing interventions, PSI conducted quantitative surveys in 2004 and 2006 to: (1) identify changes in key risk behaviors and their associated factors over time, and (2) evaluate whether those changes were associated with exposure to PSI's interventions. It is expected that the findings will contribute to a better understanding of HIV risk behaviors and effective programmatic strategies for this key population.

### PSI's kathoy-specific interventions in Laos

PSI began implementing outreach to Lao kathoy in 2002 by conducting peer-led interpersonal communication activities in a limited number of locations frequented by kathoy, with the objective of increasing condom use. PSI also launched a brand extension of the popular *Number One Deluxe *condom brand, distributed in Laos since 1999. The new product, *Number One Deluxe Plus *(NODP), was conceived partly in response to the observation that MSM and kathoy had difficulty finding affordable water-based lubricant, a desirable product that helps facilitate condom use and decrease breakage. Each NODP package contains two condoms and a sachet of water-based lubricant. Also, peer educators began to distribute an informational brochure bearing the NODP logo. The brochure explained that a condom should be used every time with every partner and that water-based lubricant should be used to prevent condom breakage. Between 2004 and 2006, PSI scaled up its interpersonal communication activities for kathoy and their partners with two goals in mind: 1) increase the availability of condoms/water-based lubricant and; 2) positively influence the target population's self-efficacy to insist on condom use with casual partners. The expanded intervention featured two drop-in centers, one in Vientiane and one in Savannakhet, which was opened near the end of the study period. PSI developed the drop-in centers as safe spaces where kathoy-specific health information and referrals to kathoy-friendly health services could be obtained. The centers also provided launching points for peer education outreach through group discussions, team-building activities, and peer education training sessions and workshops. Another component of the expanded intervention was a three-day camping trip that took place once or twice annually. Camping trip facilitators used games and other activities to provide participants with health education and life skills training related to HIV prevention issues, such as self-efficacy for negotiating condom use. Finally, in Vientiane only, social network meetings were organized once or twice annually at the drop-in center to promote the center and NODP.

## Methods

### Theoretical framework

**Figure 1:**
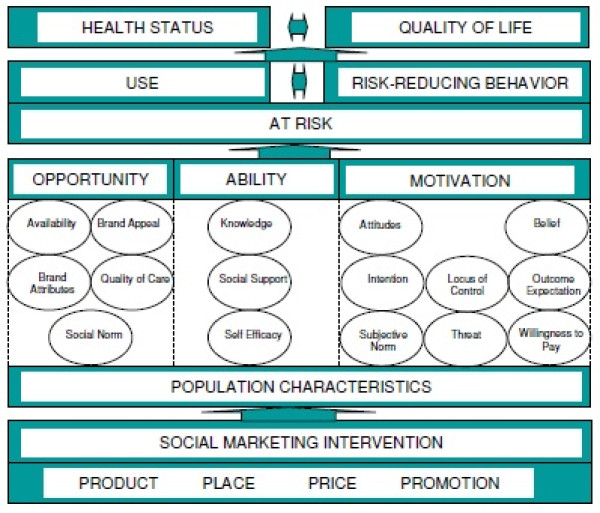
**The performance framework for social marketing (PERForM), with the PSI behavior change framework as a component of the second level**.

The study design was guided by PSI's internal frameworks for behavior change and health impact, the Performance Framework for Social Marketing (PERForM) and the PSI Behavior Change Framework (Figure 1). PERForM describes a set of theoretical pathways through which social marketing interventions can influence behaviors that have health-related consequences. The goal of the social marketing intervention is to improve the health status or quality of life of at-risk individuals by influencing behavioral constructs in ways that lead to greater use of protective products or services and/or to increased risk-reducing behavior.

The PERForM framework distinguishes between two different types of constructs associated with behaviors of interest: population characteristics and mutable factors. By targeting the constructs thought to have the strongest correlation with the behaviors of interest, a social marketing intervention can bring about changes in those behaviors, either directly or indirectly.

The second level of the PERForM framework contains categories of constructs from the PSI Behavior Change Framework. As Figure 1 indicates, these can be thought of as opportunity, ability, or motivation, a framework initially introduced by Rothschild [24]. Opportunity constructs are institutional or structural factors that affect an individual's desired behavior; ability constructs are the skills or proficiencies needed to perform the behavior; and motivation constructs influence the person**' **s desire to perform the behavior. These constructs are drawn from health behavior theories including the Health Belief Model [25] and the Theory of Reasoned Action [26], as well as marketing theory [27]. PERForM and the PSI Behavior Change Framework are described in more detail elsewhere [28].

Our study focuses on one opportunity construct, availability; two ability constructs, knowledge and self-efficacy; and three motivation constructs, attitudes, intention, and threat. Availability and self-efficacy were both found to be correlated with condom use in PSI's 2004 survey, and thus two programmatic goals were to increase the availability of condoms/water-based lubricant and to positively influence the target population's self-efficacy to insist on condom use with casual partners [29].

### Sample and design

Two quantitative surveys were conducted in November 2004 (*n *= 288) and June 2006 (*n *= 415). Respondents were aged 15-35 and were from three urban centers in Laos: Vientiane, Luang Prabang, and Savannakhet. Kathoy were eligible for inclusion in the study if they reported having oral or anal sex with a man in the past six months. We included youth under 18 after findings from the 2002 qualitative research revealed that kathoy engage in high risk behaviors starting at an early age.

The same time-location sampling strategy was used to collect data during both study rounds. Researchers conducted a mapping exercise of venues where kathoy congregated in each of the three urban centers. In 2004, a total of 144 venues were identified (94 in Vientiane, 35 in Savannakhet, and 15 in Luang Prabang). In 2006, the list of venues was updated: 139 were identified in total (102 in Vientiane, 20 in Savannakhet, and 17 in Luang Prabang). Researchers next created a list of sampling clusters that identified the time slots with the greatest number of kathoy present on weekdays and weekends for the sampling framework. Probability-proportional-to-size sampling was then used to select 36 clusters in 2004 and 67 clusters in 2006; the average cluster size in 2006 was smaller than that in 2004, which accounts for the additional clusters selected at follow-up. Fieldworkers visited each cluster at a peak time for the target population, staying for two hours on each occasion and interviewing every eligible kathoy who agreed to participate in the study.

A screening questionnaire established respondent eligibility and explained confidentiality before requesting verbal confirmation of the respondent's willingness to continue with the survey. No names, telephone numbers or other identifying information were collected. While respondents were recruited in public venues, they were allowed to complete the survey at a location of their choice, one where they felt comfortable. Surveyors were trained in safeguarding respondents' confidentiality and received sensitivity training prior to data collection. Data were stored only on the data analysts' computers and paper questionnaires were destroyed after data entry was complete.

The Laos Ministry of Health (MOH), through what is now known as the National Center for the Control of HIV/AIDS (NCCA), approved both studies. The MOH was the only active organization approving behavioral surveys in Laos on HIV/AIDS during the study years (2004-2006) and to present day.

### Measures and data collection

Eight kathoy fieldworkers and two female fieldworkers collected data from study respondents with a paper/pen survey form. The study questionnaire asked close-ended questions about background characteristics; condom and lubricant use; sexual history; sexual partners; behavioral constructs of interest; and exposure to PSI interventions. The questionnaire also distinguished between two types of sexual partners: "Boyfriends" defined as regular partners to whom kathoy are emotionally committed and "casual partners," defined as men with whom kathoy are not in an emotionally committed relationship, or men they might only have had sex with once or a few times.

The questionnaire was developed in English and then translated into Lao. It was pretested with a sample of 24 respondents at venues not selected for the survey. Kathoy who participated in the pretesting exercise were not eligible to participate in the survey. All data were collected in the Lao language and each interview took approximately 40 min to complete. Field managers ensured that participants were only interviewed once.

### Analysis

SPSS 13.0 was used for all data entry and analyses. While we conducted additional data analyses to provide programmatic direction, only a subset of results are presented in this paper to evaluate the effectiveness of PSI/Laos integrated communications approach. To identify changes in key behaviors and correlates over time, we combined data from the first and second survey rounds and ran UNIANOVA tests to identify significant differences between study years. The following sociodemographic characteristics were used as controls: province where the interview was conducted, age, highest level of education completed, occupation, average monthly income, and current living arrangements.

To evaluate whether significant changes in behavior or correlates were associated with exposure to PSI's interventions, we followed a similar strategy. We combined data from both study rounds and ran UNIANOVAs to test the effect of exposure on items that had changed significantly between 2004 and 2006. The same sociodemographic characteristics were used as controls. Baseline levels from 2004 were compared to three exposure categories in **2006: **none, low, and high. "None" was the designation for respondents who had never heard of PSI's kathoy-specific activities but may have participated in HIV prevention activities sponsored by other organizations. Those who had heard of PSI's kathoy-specific activities, read PSI's brochure for kathoy or heard of PSI's drop-in center were classified as having "low" exposure. Kathoy who had ever been contacted by a peer educator, attended a PSI discussion group, or gone on a camping/training trip were categorized as having "high" exposure.

## Results

### Baseline and follow-up sample descriptions

Table 1 presents sociodemographic characteristics for respondents who participated in the two surveys. The two study populations were similar in terms of age, education, living arrangements, and occupation. They, did, however differ significantly on reported income: incomes were higher in 2006 than they were in 2004, with significantly fewer kathoy in the lowest income category (*p *< .001) and significantly more in the middle income category (*p *= .03).

**Table 1:** Sociodemographic characteristics of kathoy surveyed in Luang Prabang, Vientiane, and Savannakhet, Laos (November 2004 and June 2006)

	2004 total (*n *= 288)		2006 total (*n *= 415)		*P*-Value
Type of transgender	n	%		%	

Transgendered MM^a^	130	45.5	197	47.5	.543

Transgendered MW^b^	158	55.5	218	52.5	.543

Age	n	%	n	%	

15-20 years old	132	45.8	193	46.5	.414

21-25 years old	87	30.2	136	32.8	.355

26-30 years old	41	14.2	61	14.7	.851

31-35 years old	28	9.7	25	6.0	.965

	Mean = 22.4 years old		Mean = 21.8 years old		.213

Education	n	%	n	%	

Never attended school	0	0	1	0.2	.405

Primary school	24	8.3	20	4.8	.059

Lower secondary school	83	28.8	133	32.0	.362

Upper secondary school	121	42.0	193	46.5	.239

Higher than secondary school	60	20.8	68	16.4	.133

Marital status	n	%	n	%	

Not married	286	99.3	412	99.3	.666

Average monthly income^c^	n	%		n	

200,000 kip or less	80	27.8	57	13.7	<.001

200,001 kip to 600,000 kip	128	44.4	209	50.4	.123

600,001 kip to 1,000,000 kip	37	12.8	79	19.0	.030

1,000,001 kip to 3,000,000 kip	34	11.8	63	15.2	.202

3,000,001 kip or higher	9	3.1	7	1.7	.209

	Mean = 741,194 kip		Mean = 812,246 kip		

Occupation	n	%		n	

Student	69	24.0	87	21.0	.348

Hairdresser/beauty salon employee	63	21.9	99	23.9	.540

Merchant/small shop owner	49	17.0	76	18.3	.658

Construction worker	13	4.5	27	6.5	.263

Waiter	13	4.5	22	5.3	.638

Guesthouse/hotel employee	8	2.8	9	2.2	.606

Teacher	7	2.4	4	1.0	.124

Government worker	6	2.1	9	2.2	.939

Army soldier	1	0.3	0	0	.230

Other	2	0.6	22	5.3	.638

Unemployed	57	19.8	60	14.5	.062

^a^Transgendered MM are defined as biological males who identify as female, but in appearance are male

^b^Transgendered MW are defined as biological males who identify as female, and in appearance are female

^c^10,000 kip is equivalent to approximately US$ 1

### Trends in behavior, correlates, and exposure to the intervention

Table 2 presents changes in protective behaviors and correlates between the two survey rounds. All three protective behaviors increased over time. Around three-quarters of kathoy in 2006 reported condom use at last anal sex with casual partners and with boyfriends, significant increases from 2004 (75.5% vs. 57.5% and 76.7% vs. 23.5%, respectively, both *p *< .001). The proportion of kathoy reporting water-based lubricant use for anal sex also increased significantly, from 56.6% in 2004 to 81.1% in 2006 (*p *< .001).

**Table 2:** Trends in behavior, correlates, and exposure to the intervention among kathoy aged 15-35 in Luang Prabang, Vientiane, and Savannakhet, Laos (November 2004 and June 2006)

Indicators	November 2004	June 2006	*P*-Value
		
	*N *= 288	*N *= 415	
Behavior/Use^a^	%	%	

Used condom with casual partner at last anal sex^b^	57.5	75.5	<.001

Used condom with boyfriends at last anal sex^c^	23.5	76.7	<.001

Ever used water-based lubricant for anal sex^d^	54.6	81.1	<.001

Opportunity	%	%	

Availability			

Condoms are available any of the places where I spend time during the day^e^	55.0	84.6	<.001

Number One Deluxe Plus is available in any of the places where I meet sex partners^f^	5.8	26.5	<.001

Number One Deluxe Plus is available in any of the places where I spend time during the day^g^	8.3	22.3	.016

Ability	%	%	

Knowledge			

Knows that oil-based lubricants increase condom breakage	33.6	9.1	<.001

Knows that having an STI can increase the likelihood of contracting HIV	87.9	75.3	<.001

Can identify at least two STI symptoms	63.5	60.5	.451

Knows that a healthy-looking person can still be infected with HIV	90.4	49.5	<.001

Knows that consistent and correct condom use with regular partner can prevent STIs^h^	89.3	97.3	<.001

Self efficacy			

Always able to insist on condom use with casual partners in the past 6 months	31.6	34.8	.453

Motivation	%	%	

Attitudes			

Believes it is very important to use condoms with regular partners to prevent HIV	58.7	91.7	<.001

Intention			

Much more likely to use condoms with casual partners if water-based lubricant available	71.3	56.1	<.001

Threat			

Very concerned about getting HIV/AIDS	71.9	71.1	.825

Exposure	%	%	

Has heard of Number One Deluxe Plus	53.4	47.6	.150

Has ever heard of PSI transgender activities	n/a	78.6	n/a

Has ever read the PSI transgender brochure	n/a	56.6	n/a

Has ever heard of the Peuan Mai drop in center	n/a	59.5	n/a

Has ever been contacted by a PSI peer educator	n/a	47.7	n/a

Has ever attended a PSI group discussion^i^	n/a	85.9	n/a

Has ever attended a PSI camping/training session^j^	n/a	48.0	n/a

^a^Control variables for each item included in the table are as follows: province where the interview was conducted, age, highest level of education completed, occupation, average monthly income in kip and current living arrangements

^b^Among respondents who had ever used a condom and had casual partners (*n *= 612). In round 1, condom use at last sex with casual partners was defined as katoy who had used a condom the last time they had anal sex with casual partners. However, in round 2, condom use at last sex with casual partners had a more stringent definition and was defined as katoy who responded that they had used a condom the last time they had anal sex from the moment of penetration to the end of intercourse with casual partners

^c^Among respondents who had ever used a condom and had a boyfriend (*n *= 455)

^d^Among respondents who had ever heard of Number One Deluxe Plus (NODP) (*n *= 485). At the time of the study, NODP was the only water-based lubricant available on the Lao market

^e^This item includes respondents who answered "all places, most places, or some places" to the following question, "Are condoms available in or near the places you spend time during the day?"

^f^This item includes respondents who answered "all or some places" to the following question, "Would you say NODP is available in or near the places where you meet your sex partners?"

^g^This item includes respondents who answered "all or some places" to the following question, "Would you say NODP is available in or near the places where you spend time during the day?"

^h^The item "knows that consistent and correct condom use with regular partner can prevent HIV" was asked in 2006, but not in 2004. Therefore, no finding for this HIV indicator is included in the above table

^i^Among those who had ever been contacted by a PSI peer educator (*n *= 198)

^j^Among those who had ever been contacted by a PSI peer educator (*n *= 198)

For opportunity-related constructs, all three items measuring perceptions of availability increased significantly. More kathoy in 2006 than in 2004 reported that condoms were available in locations where they spend time during the day (84.6% vs. 55.0% *p *< .001). There were also significant increases in perceived availability of NODP condoms in places where kathoy met sex partners (26.5% in 2006 vs. 5.8% in 2004, *p *< .001) and spent time during the day (22.3% in 2006 vs. 8.3% in 2004, *p *< .001).

In terms of ability, one knowledge item showed significant improvement over time. More kathoy in 2006 than in 2004 could identify the role of consistent and correct condom use with regular partners in preventing STIs (97.3% vs. 89.3%, *p *< .001). Unfortunately, there were significant declines in other knowledge items over time, namely that oil-based lubricants increase condom breakage (33.6% in 2004 vs. 9.1% in 2006, *p *< .001); having an STI can increase the likelihood of contracting HIV (87.9% in 2004 vs. 75.3% in 2006, *p *< .001); and that a healthy-looking person can have HIV (90.4% in 2004 vs. 49.5% in 2006, *p *< .001). There was no significant change in the proportion of kathoy able to identify at least two STI symptoms or reported self-efficacy for condom negotiation with casual partners.

For motivation-related constructs, significantly more respondents agreed with the statement, "It is very important to use condoms with regular partners to prevent HIV" (58.7% in 2004 vs. 91.7% in 2006, *p *< .001). On the other hand, the proportion of respondents agreeing that they were "much more likely to use condoms with casual partners if water-based lubricant is available" declined significantly (71.3% in 2004 vs. 56.1% in 2006, *p *< .001). There was no significant change in the level of perceived threat for contracting HIV/AIDS.

One measure of exposure to the PSI intervention was included in both study rounds, and six other measures of exposure were added in 2006. There was no significant difference between study rounds in the proportion of respondents who reported having heard of NODP. The other exposure variables measured in 2006 revealed that nearly 80% of kathoy reported that they had ever heard of PSI's kathoy-specific interventions. Slightly more than half had read the PSI brochure for kathoy (56.6%) and had heard of the New Friends (*Peuan Mai*) drop-in center in Vientiane (59.5%). Nearly half (47.7%) had been contacted by a PSI peer educator, and among them, 85.9% had attended a PSI group discussion and 48.0% had attended a PSI camping trip/training session.

### Effectiveness of PSI's kathoy-specific interventions

Table 3 shows associations between exposure to PSI kathoy-specific interventions and changes in behaviors and correlates from 2004 to 2006. Exposure had a significant overall effect on condom use at last anal sex with casual partners (*p *< .001). All three exposure groups reported significantly higher levels of condom use than the baseline group, and kathoy with high exposure were more likely to use condoms with casual partners than those with low exposure (80.3% vs. 68.0%, *p *< .05).

**Table 3:** Effectiveness of PSI's transgender-specific interventions on behavior and correlates among kathoy aged 15-35 in Luang Prabang, Vientiane, and Savannakhet, Laos (November 2004 and June 2006)

Indicators	Exposure to PSI's transgender interventions^a^				
	**Ref. (*n *= 288) 41.0%**	**None (*n *= 58) 8.3%**	**Low (*n *= 159) 22.6%**	**High (*n *= 198) 28.2%**	**Sig^b^**

Behavior/Use^c^	%	%	%	%	

Used condom with casual partners at last anal sex	57.5^a^	77.9^bd^	68.0^b^	80.3^cd^	<.001

Used condom with boyfriends at last anal sex	23.5^a^	83.7^b^	70.2^b^	79.8^b^	<.001

Ever used water-based lubricant for anal sex	54.6^a^	64.6^a, b^	76.0^b^	86.4^b^	<.001

Opportunity					

Availability	%	%	%	%	

Condoms available any of the places where spend time during the day	55.0^a^	83.2^b^	81.2^b^	87.5^b^	<.001

Number One Deluxe Plus is available in any of the places where meet sex partners	5.7^a^	55.4^b^	23.6^c^	25.4^c^	<.001

Number One Deluxe Plus is available in any of the places where spend time during the day	8.3^a^	44.4^b^	17.5^a, b^	22.9^b^	.008

Ability					

Knowledge	%	%	%	%	

Knows that oil-based lubricants increase condom breakage	33.6^a^	1.6^b^	9.3^b^	11.0^b^	<.001

Knows that having an STI can increase the likelihood of contracting HIV	87.9^a^	66.9^b^	75.0^b^	77.8^b^	<.001

Knows that a healthy-looking person can still be infected with HIV	90.5^a^	43.5^b^	46.3^b^	53.8^b^	<.001

Knows that consistent and correct condom use with regular partner can prevent STI	89.3^a^	96.2^a,b^	97.6^b^	97.4^b^	.005

Motivation					

Attitudes	%	%	%	%	

Believes it is very important to use condom with regular partners to prevent HIV	58.8^a^	94.7^b^	87.9^b^	93.9^b^	<.001

Intention	%	%	%	%	

Much more likely to use condoms with casual partners if lubricant available	71.4^a^	46.5^b^	56.3^b^	58.3^b^	.001

^a^The "baseline" category contains respondents who were surveyed in 2004. The "none" exposure category contains respondents who had never heard of PSI's transgender-specific activities. The "low" exposure category contains respondents who had ever heard of PSI transgender-specific activities, read the PSI transgender-specific brochure, or heard of the Peuan Mai transgender drop-in center. "High" exposure contains respondents who had ever been contacted by a PSI transgender peer educator, attended a PSI group discussion, or attended a PSI camping/training session

^b^The significance in the last column represents whether there was a significant effect of exposure overall

Proportions with the same letter in their superscripts do not differ significantly from one another according to the least significance difference (LSD) test

^c^Percentages are adjusted for province, age, education, occupation, average monthly income, and living situation

For "condom use with a boyfriend at last anal sex," a significant overall correlation was observed between exposure and behavior (*p *< .001). However, there were no significant differences between exposure groups, which means that the increase in the proportion of condom users at last sex with a boyfriend cannot be attributed to exposure to PSI's kathoy-specific interventions. A similar pattern was found for the behavior "ever used water-based lubricant during anal sex." There was a significant overall correlation (*p *< .001), but the low and high exposure groups did not differ significantly from each other, or from the unexposed group.

The pattern of a significant overall effect of exposure, but no difference between exposure categories was also found for several constructs. While the following constructs showed improvement over time, improvements could not be attributed to exposure to PSI's kathoy-specific interventions: "condoms available any of the places where spend time during the day," "it is very important to use condoms with regular partners to prevent HIV," and "I am much more likely to use condoms with a casual partners if lubricant is available." For the item "knows that consistent and correct condom use with a regular partner can prevent STIs," the same pattern was true. However, for all three exposure groups, over 90% of respondents were aware that consistent and correct condom use with a regular partner can prevent STIs.

Just as some positive changes could not be attributed to PSI's interventions, the same was true for the decline in the three knowledge items about oil-based lubricants, the relationship between STIs and increased risk for HIV, and the fact that a healthy-looking person can still be infected with HIV. There were no significant differences between the any of the exposure groups.

Findings about the availability of NODP in places where kathoy meet their partners and where they spend time during the day were counterintuitive. While a significant overall correlation was found in both cases (*p *< .001 and *p *< .01, respectively), the no exposure group had significantly higher levels of agreement than either one or both of the other exposure categories (*p *< .05 and *p *< .01, respectively).

## Discussion

The objectives of this study were to (1) identify changes in key behaviors and correlates related to HIV risk among Lao kathoy, and (2) evaluate whether those changes were associated with exposure to PSI's kathoy-specific social marketing interventions. Most importantly, we found that there was improvement in condom use and use of water-based lubricant over time and that exposure to PSI programming, especially speaking with a peer educator or attending group activities, was associated with a higher level of condom use at last anal sex with casual partners. Interpersonal communication appears to be a successful strategy for changing these behaviors, which is corroborated by other interventions targeting Indonesian waria that relied heavily on outreach workers and peer educators to improve condom use [30]. At the same time, the proportion of highly exposed 2006 respondents reporting condom use at last sex with casual partners was only 80.3%, indicating that more programmatic reach is needed to achieve a higher level of behavior change.

Changes in correlates were mixed. There was no significant improvement in self-efficacy over time, which merits attention. PSI should consider expanding its strategy to include activities that boost self esteem and improve kathoy's negotiation power in sexual relationships as well as their ability to negotiate condom use. The important role of these factors has been corroborated in the literature and messaging on them should be a priority [7-11]

There were, however, improvements in perceived availability of PSI's NODP condom product. After formative research in 2002 indicated that there was high demand for water-based lubricant among kathoy but that they were averse to the smell of non-fragrant lubricant, PSI launched *Number One Deluxe Plus*, which contains two *Number One *condoms and a sachet of strawberry-flavored water-based lubricant. Simply increasing product availability on the market, ensuring that it is visible in outlets, and promoting its availability through simple methods such as distributing brochures may be enough to ensure uptake of a highly desired product. More expensive and intensive interpersonal communication interventions are not necessarily merited.

Our analysis identified some other desirable trends that cannot be attributed to PSI's kathoy-specific interventions because intervention exposure did not appear to be a factor. Only 23.5% of kathoy at baseline reported using a condom with a boyfriend at last anal sex, but this behavior was reported by 83.7% of unexposed respondents, 70.2% of low-exposure respondents, and 79.8% of high-exposure respondents in 2006. Such dramatic increases, coupled with a lack of significant differences between the unexposed and exposed groups, suggest that other factors may have influenced behavior. A similar pattern, but with smaller increases, was found for the attitudinal correlate, "believes it is very important to use condoms with regular partners to prevent HIV." Both trends may reflect environmental factors not measured in the study. They may also reflect exposure to other interventions, including those addressing the general population and those tailored to MSM and kathoy. (During the study period, the Lao Youth Action AIDS Prevention Program [LYAP] and the Burnet Institute sponsored HIV prevention activities for MSM, including kathoy, in Vientiane, Champasak, and Luang Prabang.)

Concern is warranted about the significant decline between 2004 and 2006 on the intention to use condoms with casual partners if water-based lubricant is available. There was no difference among the exposure groups, which suggests that an environmental factor might have negatively affected this desirable intention. Another possibility is that while the intervention sought to convey the importance of using water-based lubricant in conjunction with condoms, individuals may have disregarded the second part of the message if it was not sufficiently emphasized.

Communicating the difference between water-based and oil-based lubricants is another key issue. In 2004, only 33.6% of respondents agreed with the statement, "Oil-based lubricants increase condom breakage." Knowledge about the dangers of using oil-based lubricants declined to 9.1% in 2006, with no significant differences between exposure categories. Taken together the findings about water-based and oil-based lubricant demonstrate that while increasing uptake of a highly desired product may be relatively simple, it is important to use interpersonal communication and accurate messaging to ensure the proper use of that product as well as to differentiate it from harmful products.

### Limitations

The following study limitations should be kept in mind. Both surveys were cross sectional and do not imply causal associations between correlates and behavior. There were some challenges during data collection. After recruiting participants at sites where kathoy congregate socially, fieldworkers were not always able to find appropriate locations to conduct the interviews, and the lack of privacy may have resulted in some information bias. Likewise, the use of kathoy interviewers could have biased study results and led to some over-reporting of desirable behaviors. While precautions were taken to ensure that the most appropriate interviewers were hired, and kathoy had said during formative research and pretesting exercises that they would prefer being interviewed by women and kathoy, they may have actually felt less comfortable providing sensitive information to a peer (kathoy).

The study population is quite young: the majority of kathoy respondents from both study rounds were under 25 years old. This may have been the result of conducting the survey in venues such as beer shops, hotels and guest houses, nightclubs, and beauty salons. Additional research may need to be conducted with older kathoy who do not congregate in these venues to identify appropriate approaches for reaching them and understanding how successful PSI's integrated communications approach has been at reducing their risk behavior.

No data about sexual partners are presented in this paper. During both study rounds, we collected data among partners aged 15 to 25 years. During formative research in 2002, kathoy reported a preference for younger male partners whom they often paid for sex. Since this behavior is largely clandestine, we were only able to draw a convenience sample of partners during both study rounds and to generate descriptive statistics [31]. If possible, subsequent surveys among kathoy should include some basic information about partners to provide additional information, such as age of partners and whether or not there was payment for sex.

The proportion of 2006 respondents who reported never having been exposed to PSI interventions was only 8.3%, which presented challenges during data analysis when constructing exposure variables; some spurious findings may have resulted, particularly for measures of perceived availability of NODP. Likewise, the study questionnaire did not ask about exposure to LYAP and Burnet activities, so no control measure could be used during evaluation analyses. Finally, there were no measures for environmental factors that may have affected changes in behavior or behavioral determinants. As a result, our ability to interpret significant differences between the reference group and those not exposed to interventions is limited. Future studies could use matching strategies, such as propensity score matching or coarsened exact matching to control for these factors during data analysis [32].

## Conclusions

This study is one of few that evaluates transgender-specific HIV prevention interventions. It demonstrated significant improvement in condom use and use of water-based lubricant among kathoy between 2004 and 2006, and showed that exposure to PSI's intervention was associated with a higher level of condom use at last anal sex with casual partners. Study findings from PSI and the Burnet Institute apparently influenced the decision by Lao authorities to include MSM in their national HIV strategy [33]. This underscores the potential for HIV prevention research and practice to have widespread policy implications and leads us to conclude that it is feasible to reach MtF transgender persons with an integrated approach that combines product promotion, peer education, and other types of interpersonal communication in countries where authorities create an enabling environment and help decrease stigma and enable safer sexual behavior.

## Competing interests

The authors declare that they have no competing interests.

## Authors' contributions

At the time of this study, KL was a Regional Researcher for PSI based in Asia. XP was the Research Manager for PSI/Laos and JC was a Technical Advisor for PSI/Laos. KL, XP, and JC developed the original study design and XP oversaw data collection. JC completed data analysis and writing for the original project report. KL reviewed the analysis and final report, and prepared the manuscript for this article. MK conducted the literature review, wrote the background for the introduction, and helped with editing. All authors read and approved the final manuscript.

## Pre-publication history

The pre-publication history for this paper can be accessed here:

http://www.biomedcentral.com/1471-2458/11/872/prepub
